# Development of Volumetric Independent Dose Calculation System for Verification of the Treatment Plan in Image-Guided Adaptive Brachytherapy

**DOI:** 10.3389/fonc.2020.00609

**Published:** 2020-05-13

**Authors:** Sang-Won Kang, Jin-Beom Chung, Kyeong-Hyeon Kim, Ji-Yeon Park, Hae-Jin Park, Woong Cho, Sven Olberg, Tae Suk Suh, Justin C. Park

**Affiliations:** ^1^Department of Biomedical Engineering, Department of Biomedicine and Health Sciences, College of Medicine, The Catholic University of Korea, Seoul, South Korea; ^2^College of Medicine, Research Institute of Biomedical Engineering, The Catholic University of Korea, Seoul, South Korea; ^3^Department of Radiation Oncology, Seoul National University Bundang Hospital, Seongnam, South Korea; ^4^Department of Radiation Oncology, University of Florida Health Proton Therapy Institute, Jacksonville, FL, United States; ^5^Department of Radiation Oncology, Ajou University School of Medicine, Suwon, South Korea; ^6^Department of Radiation Oncology, Seoul National University Boramae Medical Center, Seoul, South Korea; ^7^Department of Biomedical Engineering, Washington University in St. Louis, St. Louis, MO, United States; ^8^Department of Radiation Oncology, School of Medicine, Washington University in St. Louis, St. Louis, MO, United States

**Keywords:** independent dose calculation system, image-guided adaptive brachytherapy, American Association of Physicists in Medicine Task Group 43, dose grid, equivalent dose in 2-Gy fractions

## Abstract

**Purpose:** This study aimed to develop a volumetric independent dose calculation (vIDC) system for verification of the treatment plan in image-guided adaptive brachytherapy (IGABT) and to evaluate the feasibility of the vIDC in clinical practice with simulated cases.

**Methods:** The vIDC is based on the formalism of TG-43. Four simulated cases of cervical cancer were selected to retrospectively evaluate the dose distributions in IGABT. Some reference point doses, such as points A and B and rectal points, were calculated by vIDC using absolute coordinate. The 3D dose volume was also calculated to acquire dose-volume histograms (DVHs) with grid resolutions of 1.0 × 1.0 (G_1.0_), 2.5 × 2.5 (G_2.5_), and 0.5 × 0.5 mm^2^ (G_0.5_). Dosimetric parameters such as D_90%_ and D_2cc_ doses covering 90% of the high-risk critical target volume (HR-CTV) and 2 cc of the organs at risk (OARs) were obtained from DVHs. D_90%_ also converted to equivalent dose in 2-Gy fractions (EQD2) to produce the same radiobiological effect as external beam radiotherapy. In addition, D_90%_ was obtained in two types with or without the applicator volume to confirm the effect of the applicator itself. Validation of the vIDC was also performed using gamma evaluation by comparison with Monte Carlo simulation.

**Results:** The average percentage difference of point doses was <2.28%. The DVHs for the HR-CTV and OARs showed no significant differences between the vIDC and the treatment planning system (TPS). Without considering the applicator volume, the D_90%_ of the HR-CTV calculated by the vIDC decreases with a decreasing calculated dose-grid size (32.4, 5.65, and −2.20 cGy in G_2.5_, G_1.0_, and G_0.5_, respectively). The overall D_90%_ is higher when considering the applicator volume. The converted D_90%_ by EQD2 ranged from −1.29 to 1.00%. The D_2cc_ of the OARs showed that the averaged dose deviation is <10 cGy regardless of the dose-grid size. Based on gamma analysis, the passing rate was 98.81% for 3%/3-mm criteria.

**Conclusion:** The vIDC was developed as an independent dose verification system for verification of the treatment plan in IGABT. We confirmed that the vIDC is suitable for second-check dose validation of the TPS under various conditions.

## Introduction

Image-guided adaptive brachytherapy (IGABT) based on magnetic resonance images (MRIs) has been introduced as a new standard technique to improve the treatment outcome in cervical cancer (1–6). IGABT delivers a high dose with a small number of fractions after external beam radiotherapy (EBRT). The treatment plan of IGABT is optimized by using dosimetric parameters to meet dose constraints for the organs at risk (OARs) and high-risk clinical target volume (HR-CTV). The dose constraints are normalized to the equivalent dose in 2-Gy fractions (EQD2) to produce the same radiobiological effect as in EBRT. Furthermore, IGABT based on MRIs is more advantageous in the delineation of a region of interest (ROI) such as the HR-CTV and OARs than is high-dose-rate brachytherapy (BT_HDR_) based on computed tomography (CT) images (7, 8). Because compensation of IGABT for treatment outcomes is difficult, it is more important to generate the correct treatment plan and to deliver accurate doses than with other conventional treatments (9). Thus, at each fraction, IGABT requires re-optimization of the treatment plan and re-defining of the ROIs. Furthermore, the re-optimized treatment plan should be validated for safe and accurate delivery (10).

In general, verification of treatment plan in brachytherapy is performed by comparing the point doses calculated using the treatment planning system (TPS) and an independent dose calculation (IDC) system (11, 12). In the previous studies, various IDC systems were reported (13–16). Formalism of the American Association of Physicists in Medicine Task Group 43 (AAPM TG-43) is usually used to calculate the point doses via templates or were spreadsheet based in these systems. However, these point-dose comparisons alone were not sufficient for the verification of IGABT.

For better validation in IGABT, dosimetric parameters should be calculated using an IDC system. Some commercialized IDC systems, such as BrachyCheck (ROS, California, USA) and DIAMOND for Brachytherapy (PTW, Freiburg, Germany), were recently introduced. BrachyCheck can only calculate a dose-volume histogram (DVH). In addition, DIAMOND calculates point dose and 3D dose distributions, but in order to perform 3D analysis, the dose file must be exported to other analysis software. To solve inconveniences in these commercialized IDC systems, Xianliang et al. reported dose verification software (DVS) that can calculate the point dose and the dosimetric parameters (17). The DVS also utilizes the gamma evaluation, but there are some limitations. In the DVS, the calculation method for the direction of the source is inaccurate in the ring applicator because they defined the source vectors by connecting the existing source dwell-position and next source dwell-position. Moreover, it can use only fixed dose-grid size (1.0 × 1.0 mm^2^). Therefore, we aimed to develop a volumetric IDC (vIDC) tool suitable for IGABT verification. In the vIDC, the method of determining the direction of the source will be improved, and various dose-grid sizes will be available for selection. We will also add some functions to the vIDC. Firstly, the dosimetric parameters of the HR-CTV will allow the calculation of two types (HR-CTV_W_ or HR-CTV_W/O_) with or without applicator volume, and secondly, the calculated 3D dose volumes will be able to be convert to EQD2 to account for the radiobiological effect caused by EBRT.

## Materials and Methods

### Volumetric Independent Dose Calculation System

**Figure 1:**
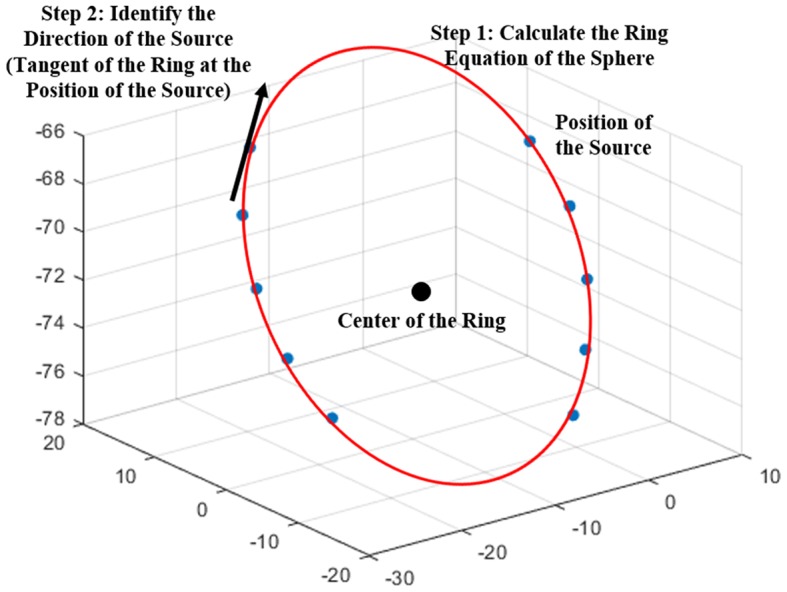
Coordinate system for determining the source direction.

In brachytherapy, it is important to accurately calculate the source position and orientation to improve the accuracy of dose calculations for tandem-and-ring applicators. The vIDC can calculate the appropriate direction of sources in the ring applicator, as shown in Figure 1. To calculate the source direction for the ring applicator, the vIDC obtains various plane vectors of the ring by using three sources' positions. Then, the vIDC selects an appropriate plane vector with analysis of the root mean square error (RMSE) between each source's position and the calculated plane vector. The ring equation of the sphere is obtained using the source positions and the selected plane vector (step 1). The vector direction of each source is calculated by using the tangent between the obtained ring equation and source position (step 2).

The dose calculation for the vIDC was based on the AAPM TG-43U1 formalism. The formalism is classified as a point and line source model, according to the method of source modeling. The line source model was used in the vIDC, and the equation of the model was as follows:

(1) $$\begin{array}{r} D\left(r,\;\theta \right)=S_{K}\cdot \Lambda \cdot \frac{G_{L}\left(r,\;\theta \right)}{G_{L}\left(r_{0},\;\theta_{0}\right)}\cdot g_{L}\left(r\right)\cdot F\left(r,\;\theta \right) \end{array}$$

where *r* is the distance from the center of an active source to a point of interest and θ is the polar angle between the source longitudinal axis and the line that connects the center of the active source and the point of interest. The *r*_0_ and the θ_0_ denote the reference distance (1 cm) and angle (90°), respectively. The air kerma strength (unit: cGy·cm^2^·h^−1^, 1 U = 1 cGy·cm^2^·h^−1^), dose-rate constant (unit: cGy·h^−1^·U^−1^), geometry factor, radial-dose function, and anisotropy function are represented as *S*_*K*_, Λ, *G*(*r*, θ), *g*(*r*), and *F*(*r*, θ), respectively. Each value of *S*_*K*_ and Λ is defined in the TPS, while *g*(*r*) and *F*(*r*, θ) are provided by the manufacturer of the source. The default grid size of the vIDC was 1.0 × 1.0 mm^2^ grid (G_1.0_). It is also possible to select various grid sizes, such as 2.5 × 2.5 (G_2.5_) and 0.5 × 0.5 (G_0.5_) mm^2^ grids.

The calculated dosimetric parameters are converted to EQD2 parameters for evaluating the radiobiological effect of the treatment plan in conjunction with EBRT. EQD2 is calculated as follows:

(2) $$\begin{array}{rcl} \text{EQD}2 & = & \;\frac{BED}{1+\frac{2}{\alpha /\beta }} \end{array}$$

(3) $$\begin{array}{rcl} \text{BED} & = & nd\left[1+\frac{d}{\alpha /\beta }\right] \end{array}$$

A biologically effective dose (BED) is obtained for calculating EQD2. The BED is used for isoeffective dose calculations, which means that the true biological dose delivered is measured. Each *n* and *d* is presented as the number of fractions and dose per fraction, respectively. The α/β ratio means the ratio of “intrinsic radiosensitivity” to “repair capability” of a specified tissue, and it was presented in this paper as the tumor (α/β ratio = 10) and OARs (α/β ratio = 3).

### Treatment Planning of Image-Guided Adaptive Brachytherapy Simulated Cases

In order to examine the efficiency of the functions and the clinical feasibility for the vIDC, we retrospectively evaluated with simulated cases treated using IGABT. Four simulated cases with squamous cell carcinoma of the cervix were treated with IGABT using an ^192^Ir HDR source and adapted a tandem-and-ring technique. These cases were approved by the Institutional Review Board (IRB). Treatment plans were generated with the Oncentra Brachy (Elekta, Stockholm, Sweden) TPS, and the 3D dose volume was calculated with a grid size of 1.0 × 1.0 mm^2^.

As in EBRT, the dosimetric parameters are used as criteria of the treatment plan (Table 1). The criteria are defined as the doses covering 90% of the HR-CTV and 2-cc volumes of each OAR (D_90%_ and D_2cc_, respectively). All plans were re-optimized with appropriate dose criteria according to the variation in anatomy and applicator location before treatment.

**Table 1:** The dose criteria used in the simulated IGABT cases.

**Structure**	**Criteria**
HR-CTV	D_90%_ = 550 cGy
Bladder	D_2cc_ <460 cGy
Rectum	D_2cc_ <420 cGy
Sigmoid	D_2cc_ <420 cGy

*IGABT, image-guided adaptive brachytherapy; HR-CTV, high-risk critical target volume*.

The point doses at points A and B and the rectum were also calculated to verify the accuracy of dose calculation using the vIDC by comparison with results calculated by the TPS. Point A is defined as 2.0 cm superior to the lateral vaginal fornix and 2.0 cm lateral to the cervical canal. Point B indicates a position 2.0 cm superior to the ring surface and 5.0 cm lateral to the midline. Point A represents dose limits that are delivered to the uterine cervix, whereas point B is used to evaluate the lateral spread of the effective doses, such as nearby doses in the pelvic wall and obturator node. The rectal point dose represents a delivered dose in the rectum. The reference point dose of the TPS was compared with that of the vIDC by using the percentage difference (%diff).

### Monte Carlo Simulation for Validation of Volumetric Independent Dose Calculation System

Validation of vIDC was performed with Monte Carlo (MC) simulations using GATE (Geant4 Application for Tomographic Emission, Version 8.1). The grid size in MC was 1.0 × 1.0 mm^2^, and the ^192^Ir source (HDR ^192^Ir mHDR-v2) has been modeled, as described by Granero et al. (18). A virtual treatment plan that included a tandem-and-ring technique was established within the simulated water phantom. The plan was imported into the TPS and the vIDC, and the respective calculation engine was used to obtain the dose distribution in the axis plane of the ring applicator's center. With gamma evaluation, dose distributions calculated by the TPS and the vIDC were compared with the MC results.

## Results

### Validation of Volumetric Independent Dose Calculation by Comparing Dose Distribution With Monte Carlo and Treatment Planning System

**Figure 2:**
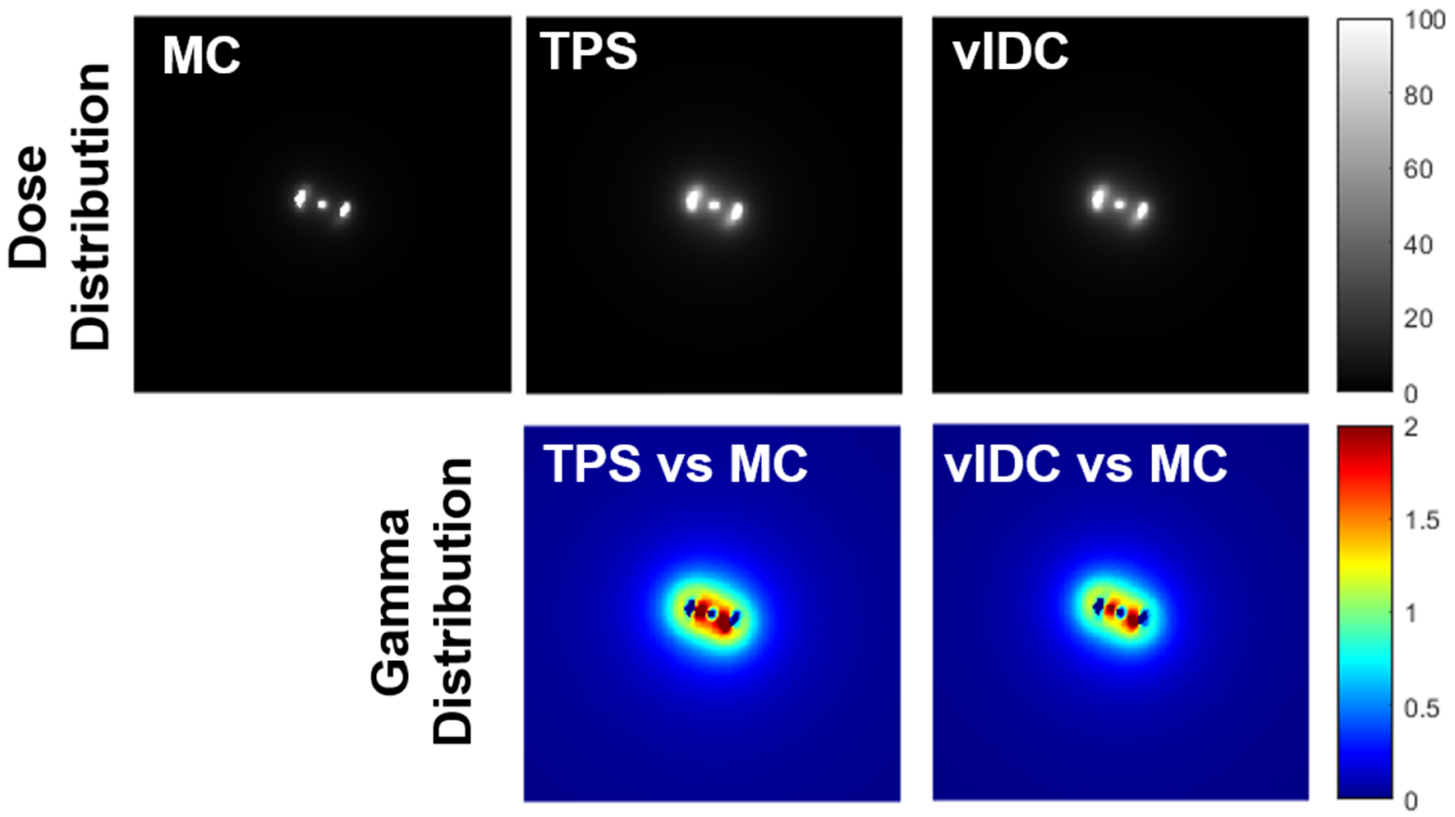
All dose distributions calculated by Monte Carlo (MC), treatment planning system (TPS), and volumetric independent dose calculation (vIDC) system. These dose distributions were analyzed with gamma evaluation using 3%/3-mm criteria.

Figure 2 shows all dose distributions calculated by MC, TPS, and vIDC. These distributions were normalized with the maximum dose. With gamma analysis, the passing rates of TPS and vIDC were 98.25% and 98.81% for 3%/3-mm criteria, respectively, through comparison with the calculated dose distribution by MC.

### Comparison of Point Doses at the Reference Points

Table 2 shows the percentage differences in the calculated point doses between TPS and vIDC at the reference points to evaluate the accuracy of the developed vIDC. All average percentage differences of vIDC were < -2.28% at reference points of the same absolute coordinates, compared with the clinical TPS.

**Table 2:** The calculated point doses and percentage differences by using TPS and vIDC for five reference points.

**Reference points**	**Point dose**		**Percent difference (TPS vs. vIDC)**
	**TPS (mean ± std, Gy)**	**vIDC (mean ± std, Gy)**	
Right point A	4.69 ± 1.03	4.67 ± 1.03	−0.40
Left point A	4.71 ± 1.03	4.69 ± 1.02	−0.39
Right point B	1.19 ± 0.30	1.16 ± 0.30	−2.00
Left point B	1.05 ± 0.32	1.03 ± 0.32	−2.28
Rectum	1.99 ± 0.33	1.97 ± 0.32	−1.01

*TPS, treatment planning system; vIDC, volumetric independent dose calculation*.

### Comparison of the Dose-Volume Histogram and Dosimetric Parameters

**Figure 3:**
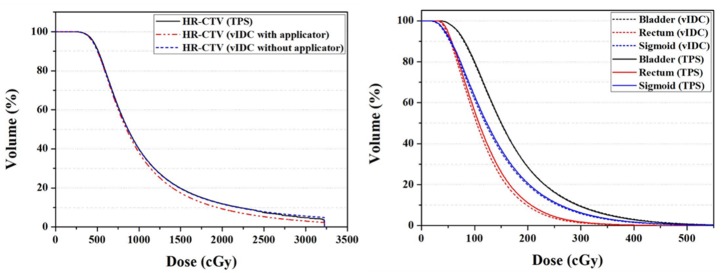
Averaged DVHs of HR-CTV (left) and OARs (right) from vIDC and TPS using 1.0-mm dose-grid size (G_1.0_). DVHs, dose-volume histograms; HR-CTV, high-risk critical target volume; OARs, organs at risk; vIDC, volumetric independent dose calculation; TPS, treatment planning system.

The vIDC facilitated comprehensive 3D dose evaluations for clinical use, even when the G_1.0_ was used. Figure 3 shows averaged DVHs from the calculated 3D dose volumes by the vIDC and TPS using the same dose-grid size of 1.0 mm (G_1.0_). As shown in Figure 3, no dose differences were observed for the HR-CTV_W/O_, bladder, and sigmoid in the DVHs. However, a dose difference with a high-dose range exceeding 1,000 cGy occurred in HR-CTV_W_. In addition, a dose difference of <10 cGy was observed at the same volume in the intermediate dose range for the rectum.

Table 3 shows the dosimetric parameters between the vIDC and TPS for the HR-CTV with or without the applicator volume for various dose-grid sizes. The volumes of the HR-CTV calculated by the vIDC were decreased by decreasing the dose-grid size, as shown in Table 3. The target volume calculated with G_2.5_ was larger in the vIDC than TPS. The minimum absolute percentage difference for the target volume was 1.43% in HR-CTV_W/O_ calculated with G_1.0_. The percentage difference for the dosimetric parameter also decreases by decreasing the dose-grid size. For the G_2.5_, the differences of D_100%_ and D_90%_ were more than −6%. The differences decreased to −3.42 and −0.44% when G_0.5_ was used. The standard deviations for both evaluated dosimetric parameters were within 40 cGy for D_100%_ and 23 cGy for D_90%_. Overall, the standard deviations were similar for the vIDC and TPS regardless of grid sizes. In addition, the D_100%_ values of vIDC were the same in HR-CTV_W_ and HR-CTV_W/O_. However, all standard deviations of D_90%_ used the same grid size were higher in HR-CTV_W/O_ than HR-CTV_W._ The differences of D_90%_ were also greater in HR-CTV_W/O_.

**Table 3:** The percentage difference of dose and volume for the HR-CTV between vIDC using three dose grid sizes with or without the applicator volume and TPS using G_1.0_.

	**Grid size**	**Target volume**		**Dosimetric parameter**			
				**D**_****100%****_		**D**_****90%****_	
		**Mean (cm^**3**^)**	**Absolute percentage difference**	**Mean ± std (cGy)**	**Percentage difference**	**Mean ± std (cGy)**	**Percentage difference**
TPS	G_1.0_	30.45	-	316.79 ± 39.05	-	525.90 ± 23.19	
vIDC with the applicator volume	G_2.5_	32.69	|7.26|	295.53 ± 38.73	−6.76	490.85 ± 20.64	−6.94
	G_1.0_	29.31	|4.17|	304.00 ± 39.20	−4.09	517.50 ± 21.53	−1.83
	G_0.5_	28.32	|7.56|	306.53 ± 39.96	−3.42	525.25 ± 21.97	−0.36
vIDC without the applicator volume	G_2.5_	33.49	|10.23|	295.53 ± 38.73	−6.76	493.50 ± 23.19	−6.14
	G_1.0_	30.02	|1.43|	304.00 ± 39.20	−4.09	520.25 ± 21.71	−1.06
	G_0.5_	29.01	|4.91|	306.53 ± 39.96	−3.42	528.10 ± 22.28	−0.44

*HR-CTV, high-risk critical target volume; vIDC, volumetric independent dose calculation; TPS, treatment planning system*.

Table 4 indicates the dosimetric parameters and volumes calculated with the TPS and vIDC for all OARs such as the bladder, rectum, and sigmoid under the condition of various dose-grid sizes. All OARs were acceptable for the D_2cc_ dose criterion shown in Table 1. For the calculated volumes for all OARs, the smallest difference (<1.82%) compared with that in vIDC and TPS was observed in the G_1.0_ used in TPS. The volume differences for all OARs in the other grid were more than 4% for G_0.5_ and 8% for G_2.5_. Compared with the dosimetric parameters of TPS using G_1.0_, all evaluated parameters of vIDC for the bladder and sigmoid have dose differences within ~-3% for G_0.5_ and G_1.0_, but there was a difference of more than −4% for G_2.5_. On the contrary, there was a dose difference for the rectum of < -3% for G_2.5_ and more than −4% for G_0.5_ and G_1.0_.

**Table 4:** The percentage differences of doses and volumes for the OARs between vIDC using three dose grid sizes and TPS using G_1.0_.

	**Grid size**	**Dosimetric parameter**	**OARs (percent difference compared with the TPS)**		
			**Bladder**	**Rectum**	**Sigmoid**
			**(Mean ± std)**	**(Mean ± std)**	**(Mean ± std)**
TPS	G_1.0_	D_10cc_	222.15 ± 75.24	132.80 ± 67.19	252.25 ± 62.63
		D_2cc_	324.25 ± 101.81	195.85 ± 85.42	367.40 ± 76.61
		D_0.1cc_	448.45 ± 133.95	271.10 ± 117.63	543.30 ± 138.11
		Mean volume of OARs (cm^3^)	57.75	45.28	168.69
vIDC	G_2.5_	D_10cc_	232.25 ± 75.45	134.4 ± 66.67	259.50 ± 62.75
			(4.34)	(2.25)	(3.08)
		D_2cc_	343.85 ± 110.17	197.60 ± 86.65	381.90 ± 76.97
			(5.65)	(0.92)	(4.14)
		D_0.1cc_	487.80 ± 150.73	276.75 ± 120.89	564.30 ± 131.48
			(8.18)	(1.95)	(4.43)
		Mean volume of OARs (cm^3^)	62.25	49.26	180.49
			|8.71|	|10.41|	|8.12|
	G_1.0_	D_10cc_	222.40 ± 76.29	127.40 ± 64.59	248.20 ± 61.02
			(−0.11)	(−4.04)	(−1.55)
		D_2cc_	327.70 ± 104.35	188.15 ± 81.57	363.35 ± 73.75
			(1.12)	(−3.82)	(−0.97)
		D_0.1cc_	458.60 ± 139.53	260.35 ± 112.72	532.90 ± 125.73
			(1.97)	(−3.89)	(−1.53)
		Mean volume of OARs (cm^3^)	57.03	44.83	165.94
			|1.43|	|1.12|	|1.82|
	G_0.5_	D_10cc_	219.60 ± 75.45	125.15 ± 64.20	244.85 ± 60.48
			(−1.36)	(−6.20)	(−2.90)
		D_2cc_	322.85 ± 102.68	185.20 ± 80.04	358.05 ± 73.34
			(−0.63)	(−5.26)	(−2.46)
		D_0.1cc_	450.60 ± 136.32	255.50 ± 110.38	523.65 ± 124.75
			(0.29)	(−5.62)	(−3.30)
		Mean volume of OARs (cm^3^)	55.48	43.53	161.51
			|4.42|	|4.53|	|4.81|

*OARs, organs at risk; vIDC, volumetric independent dose calculation; TPS, treatment planning system*.

### Comparison of the Equivalent Dose in 2 Gy per Fraction

In order to evaluate the radiobiological effect, the EQD2 of dosimetric parameters for the HR-CTV and OARs was analyzed in each faction. Table 5 shows the EQD2 for the HR-CTV and OARs in each fraction for one simulated case. For HR-CTV_W/O_, EQD2 differences in D_90%_ calculated using the vIDC and TPS ranged from −1.00 to 1.29%. For each fraction, the differences of EQD2 for dosimetric parameters were higher in OARs than HR-CTV_W/O_. The maximum difference of EQD2 was −9.15% in the sigmoid and was observed in D_0.1cc_ of the first fraction.

**Table 5:** Equivalent dose in 2-Gy fractions (EQD2) in dosimetric parameters for HR-CTV and OARs in each fraction.

	**Target**	**Dosimetric parameter**	**Equivalent dose in 2-Gy fractions (Gy) (percentage difference)**					
			**1st fraction**	**2nd fraction**	**3rd fraction**	**4th fraction**	**5th fraction**	**Total**
TPS	HR-CTV	D_90%_	6.76	6.86	6.83	6.60	6.62	33.67
	Bladder	D_0.1cc_	2.14	2.17	3.18	6.14	2.55	16.18
		D_2cc_	1.29	1.34	2.23	4.04	1.73	10.63
	Rectum	D_0.1cc_	1.69	2.06	1.34	8.34	1.06	14.49
		D_2cc_	1.17	1.35	1.00	4.39	0.74	8.65
	Sigmoid	D_0.1cc_	18.10	7.26	6.25	11.29	6.37	49.27
		D_2cc_	9.60	4.53	4.35	6.46	3.44	28.39
vIDC without the applicator volume (G_1.0_)	HR-CTV	D_90%_	6.72	6.79	6.77	6.64	6.71	33.63
			(−0.51)	(−1.00)	(−0.76)	(0.52)	(1.29)	(−0.11)
	Bladder	D_0.1cc_	2.11	2.10	3.13	6.30	2.59	16.23
			(−1.34)	(−3.31)	(−1.60)	(2.63)	(1.83)	(0.35)
		D_2cc_	1.26	1.29	2.20	4.10	1.73	10.57
			(−2.73)	(−3.55)	(−1.31)	(1.41)	(0.00)	(−0.52)
	Rectum	D_0.1cc_	1.62	1.99	1.29	7.82	1.00	13.72
			(−3.84)	(−3.42)	(−3.55)	(−6.26)	(−6.14)	(−5.32)
		D_2cc_	1.13	1.29	0.97	4.12	0.70	8.21
			(−3.86)	(−4.41)	(−3.22)	(−6.18)	(−5.23)	(−5.17)
	Sigmoid	D_0.1cc_	16.44	6.80	6.00	10.65	5.84	45.74
			(−9.15)	(−6.40)	(−4.03)	(−5.62)	(−8.27)	(−7.17)
		D_2cc_	8.99	4.27	4.18	6.21	3.25	26.90
			(−6.40)	(−5.65)	(−4.02)	(−3.96)	(−5.57)	(−5.26)

*HR-CTV, high-risk critical target volume; OARs, organs at risk*.

## Discussion

The source direction determined by Xianliang et al. (17) was obtained by connecting each source's position. This method is not suitable for obtaining accurate source direction, especially in using ring applicators. Therefore, we initially calculated the sphere's ring equation, and then we calculated the source direction using the tangent of the sphere equation at each source position. We showed the point doses in reference points and the doses in dosimetric parameters calculated by Xianliang's method in Tables 6, 7. For five reference points, the point doses using the vIDC were similar with those obtained with by Xianliang's method. In addition, the doses of dosimetric parameters calculated by Xianliang's method and the vIDC using G_1.0_ show significant differences, as shown in Table 7. Overall, the dose percentage differences of dosimetric parameters are higher in Xianliang's method than vIDC. In particular, the percentage difference in D_90%_ increased ~3 times (1.83% for the vIDC and to 5.55% for Xianliang's method) compared with the dose calculated with TPS. The percentage differences also increased with the other dosimetric parameters for OARs. However, the percentage difference compared with TPS was found to be smaller in vIDC than Xianliang's method. Thus, we can conclude that the tangential method used in this study is more accurate than Xianliang's method. Maybe this method can be applied to conventional brachytherapy.

**Table 6:** The calculated point doses and percentage differences by using TPS and Xianliang's method for five reference points.

**Reference points**	**Point dose**		**Percent difference (TPS vs. Xianliang's method)**
	**TPS (mean ± std, Gy)**	**Xianliang's method (mean ± std, Gy)**	
Right point A	4.69 ± 1.03	4.67 ± 1.03	−0.41
Left point A	4.71 ± 1.03	4.70 ± 1.02	−0.41
Right point B	1.19 ± 0.30	1.16 ± 0.30	−2.02
Left point B	1.05 ± 0.32	1.03 ± 0.32	−2.33
Rectum	1.99 ± 0.33	1.97 ± 0.32	0.87

*TPS, treatment planning system*.

**Table 7:** The dose and percentage differences in dosimetric parameters for HR-CTV and OARs by using Xianliang's method and TPS.

	**Grid size**		**HR-CTV (mean ± std, cGy)**		**Bladder (mean ± std, cGy)**	**Rectum (mean ± std, cGy)**	**Sigmoid (mean ± std, cGy)**
			**(Percent difference)**		**(Percent difference)**	**(Percent difference)**	**(Percent difference)**
TPS	G_1.0_	D_100%_	316.79 ± 39.05	D_10cc_	222.15 ± 75.24	132.80 ± 67.19	252.25 ± 62.63
		D_90_	525.90 ± 23.19	D_2cc_	324.25 ± 101.81	195.85 ± 85.42	367.40 ± 76.61
				D_0.1cc_	448.45 ± 133.95	271.10 ± 117.63	543.30 ± 138.11
Xianliang's method	G_1.0_	D_100%_	277.9 ± 72.50	D_10cc_	230.80 ± 78.75	125.2 ± 62.54	244.45 ± 59.65
			(8.26)		(3.71)	(5.39)	(2.93)
		D_90%_	500.25 ± 23.72	D_2cc_	343.20 ± 109.33	183.85 ± 77.81	359.30 ± 73.18
			(5.55)		(5.54)	(5.61)	(1.98)
				D_0.1cc_	488.05 ± 150.16	252.00 ± 105.00	524.80 ± 126.38
					(8.37)	(6.38)	(2.80)

*HR-CTV, high-risk critical target volume; OARs, organs at risk; TPS, treatment planning system*.

Compared with the calculated point dose with TPS, the calculated point doses with vIDC at the right and left point B were the dose percentage difference of more than −1%. However, these differences cannot be defined as the inaccuracy of dose calculation by vIDC, because there was a small difference in the calculated doses between the vIDC and TPS. The average dose differences between vIDC and TPS for all reference points were <0.03 Gy. Therefore, it is indicated that the dose calculations obtained with the vIDC are accurate on the basis of the dose differences.

For the effect of grid size, the percentage difference in doses of all dosimetric parameters for OARs was the smallest in G_1.0_ used in TPS. On the other hand, D_100%_ and D_90%_ for the target volume had the smallest percentage difference with G_0.5_ regardless of whether applicator volume was used. The difference is probably influenced by the dose gradient of the contoured ROI. As the dose-grid size decreases, accurate dose verification is possible, especially in regions with steep dose gradients. However, the dose difference between G_1.0_ and G_0.5_ is relatively small (<10 cGy) and can be neglected. Moreover, when the dose-grid size becomes two times smaller, the time required for dose calculation increases four times. Therefore, we recommend determining the dose-grid size, taking into account the desired dose calculation accuracy and existing time constraints.

In this study, we noted the dosimetric difference due to the applicator volume. Potter et al. (19) reported that the applicator volume does not affect the dosimetric parameters if the target volume is sufficiently larger than the applicator volume. However, the authors also mentioned that this dosimetric effect of the applicator volume needs to be investigated because it has not been clearly established. Thus, we demonstrated the dosimetric effect with and without applicator volume using the vIDC, and there was almost no the dosimetric effect, as shown in Table 3. The dose differences were <4 cGy in all D_90_. On the basis of our results, we confirmed that the dosimetric effect does not need to be considered when the volume of the HR-CTV is sufficiently large, such as cervical cancer cases.

IGABT is known to improve treatment efficiency by delivering additional dose to local lesion control after EBRT. The IGABT is usually used at high doses (D_90%_ of HR-CTV > 550 cGy) in low fractions (<5 fractions) using the ^192^Ir source with an average energy of 0.38 MeV. In contrast, the EBRT delivers normally the total dose of 45 Gy in 1.8 Gy per fraction delivered using a high-energy photon beam (20, 21). Because of the characteristic difference of these delivery doses, it is difficult to accurately determine the same radiobiological effect between IGABT and EBRT for treatment dose. Therefore, the prescribed doses in each treatment were determined by conversion to EQD2 to enable accurate optimization of the delivered doses. In our study, radiobiological evaluation was performed by calculating EQD2, and this evaluation will enable safer and more accurate treatment.

Gamma evaluation analyzed with MC simulation results was able to verify the vIDC. The gamma passing rate has no difference, although slightly better in vIDC than TPS. The point dose and dosimetric parameters of the vIDC indicated similarities with the TPS. In view of these results, we confirmed that the vIDC has almost the same performance as TPS. Thus, our vIDC can be used to validate the treatment plan as a second dose check that re-calculates the treatment plan and detects errors in the TPS. In this study, however, the vIDC was only evaluated in the tandem-and-ring cases. We will validate this vIDC in other treatment cases for clinical application in a future study.

## Conclusion

The vIDC was developed for use as a second dose check to verify the clinical IGABT treatment plans. This study revealed that vIDC has the potential to verify the dose volumes calculated by TPS. In addition, we have been found that the doses in the dosimetric parameters are more affected by the dose grid size, not the applicator volume. Finally, the vIDC can improve the safety of IGABT by verifying the treatment plans with a variety of conditions.

## Data Availability Statement

All datasets generated for this study are included in the article/supplementary material.

## Author Contributions

J-BC, TS, and JP supervised the project. S-WK and JP conceived and designed the experiments. J-YP, H-JP, and J-BC contributed the simulated cases. S-WK, K-HK, and WC built the in-house software. S-WK, SO, and JP wrote the manuscript.

## Conflict of Interest

The authors declare that the research was conducted in the absence of any commercial or financial relationships that could be construed as a potential conflict of interest.
